# 2-(5-Bromo-2-methyl­phen­yl)propan-2-ol

**DOI:** 10.1107/S1600536810042698

**Published:** 2010-10-30

**Authors:** Hui Zeng, Xin-Lin Liu

**Affiliations:** a Department of Pathology, Chinese People’s Armed Police Corps Hospital of Qinghai Province, Xining 810000, People’s Republic of China; b Institute of Cardiovascular Disease, Pingjin Hospital, Medical College of Chinese People’s Armed Police Forces, Tianjin 300162, People’s Republic of China

## Abstract

The title compound, C_10_H_13_BrO, crystallizes with four independent mol­ecules of similar geometry in the asymmetric unit. The crystal packing is stabilized by inter­molecular O—H⋯O hydrogen bonds, which link the mol­ecules into tetra­mers.

## Related literature

The title compound is an inter­mediate for the synthesis of SGLT2 inhibitors, which possess potent anti­hyperglycemic activity, see: Gao *et al.* (2010 ▶); Meng *et al.* (2008 ▶); Wang *et al.* (2010 ▶).
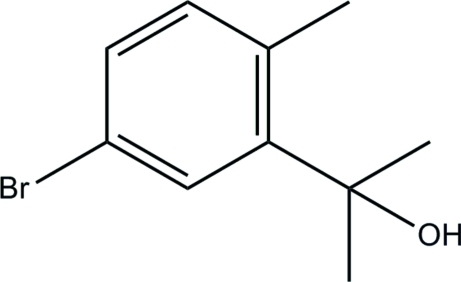


## Experimental

### 

#### Crystal data

C_10_H_13_BrO
*M* *_r_* = 229.11
Triclinic, 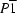

*a* = 12.074 (2) Å
*b* = 12.115 (2) Å
*c* = 15.242 (3) Å
α = 109.51 (3)°
β = 103.52 (3)°
γ = 90.70 (3)°
*V* = 2033.2 (7) Å^3^
*Z* = 8
Mo *K*α radiation
μ = 4.00 mm^−1^
*T* = 113 K
0.26 × 0.20 × 0.18 mm

#### Data collection

Rigaku Saturn CCD area-detector diffractometer
Absorption correction: multi-scan (*CrystalClear*; Rigaku, 2007 ▶) *T*_min_ = 0.423, *T*_max_ = 0.533
20930 measured reflections
7162 independent reflections
5048 reflections with *I* > 2σ(*I*)
*R*_int_ = 0.077

#### Refinement

*R*[*F* ^2^ > 2σ(*F* ^2^)] = 0.049
*wR*(*F* ^2^) = 0.102
*S* = 0.94
7162 reflections
450 parameters
H-atom parameters constrained
Δρ_max_ = 1.36 e Å^−3^
Δρ_min_ = −1.06 e Å^−3^

### 

Data collection: *CrystalClear* (Rigaku, 2007 ▶); cell refinement: *CrystalClear*; data reduction: *CrystalClear*; program(s) used to solve structure: *SHELXS97* (Sheldrick, 2008 ▶); program(s) used to refine structure: *SHELXL97* (Sheldrick, 2008 ▶); molecular graphics: *SHELXTL* (Sheldrick, 2008 ▶); software used to prepare material for publication: *SHELXTL*.

## Supplementary Material

Crystal structure: contains datablocks I, global. DOI: 10.1107/S1600536810042698/rz2503sup1.cif

Structure factors: contains datablocks I. DOI: 10.1107/S1600536810042698/rz2503Isup2.hkl

Additional supplementary materials:  crystallographic information; 3D view; checkCIF report

## Figures and Tables

**Table 1:** Hydrogen-bond geometry (Å, °)

*D*—H⋯*A*	*D*—H	H⋯*A*	*D*⋯*A*	*D*—H⋯*A*
O1—H1⋯O2^i^	0.84	1.90	2.742 (4)	179
O2—H2⋯O4^ii^	0.84	1.91	2.739 (4)	170
O3—H3⋯O1^iii^	0.84	1.90	2.727 (4)	167
O4—H4⋯O3^iv^	0.84	1.90	2.740 (4)	179

Symmetry codes: (i) 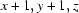; (ii) 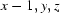; (iii) 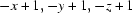; (iv) 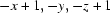.

**Fig. 1.**
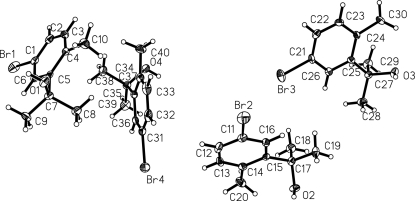
The molecular structure of the title compound, with atom labels and 40% probability displacement ellipsoids for non-H atoms.

**Fig. 2.**
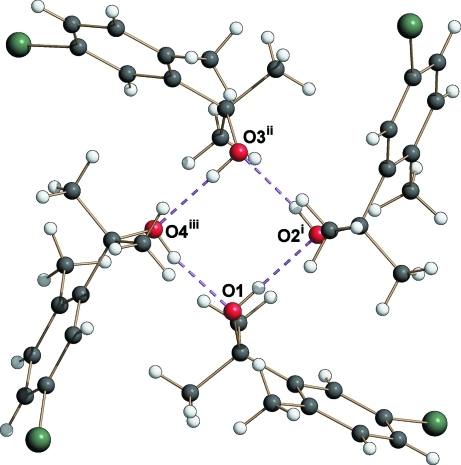
Top view of the tetramer formed by intermolecular O—H···O hydrogen bonds (dashed lines). Symmetry codes: (i) 1+x, 1+y, z; (ii) 1-x, 1-y, 1-z; (iii) x, 1+y, z.

## supplementary crystallographic information

### Comment

The title compound, whose crystal structure is reported herein, is an important intermediate for the synthesis of SGLT2 inhibitors possessing potent antihyperglycemic activity (Gao *et al.*, 2010; Meng *et al.*, 2008; Wang *et al.*, 2010).

The asymmetric unit of the title compound consists of four independent molecules of similar geometry (Fig. 1). All bond lengths and angles are not unusual. In the crystal packing, the independent molecules are linked by intermolecular O—H···O hydrogen bonds into tetramers (Table 1).

### Experimental

A dried 100-ml round-bottomed flask was charged with 2.43 g (10 mmol) of ethyl 5-bromo-2-methylbenzoate, 30 ml of dried THF and a magnetic bar, flushed with nitrogen and sealed with rubber septum. The flask was cooled with an ice-water bath, then stirring was initiated. Into the flask was slowly added 7.0 ml (21 mmol; 3.0 *M* in THF) of methyl magnesium chloride through syringe. After addition, the reaction mixture was stirred at this temperature for half an hour. The reaction mixture was poured into 200 ml of cooled water followed by addition of 200 ml of dichloromethane, and the reaction mixture was stirred and filtered off through a pad of celite. The filtrate was collected and the organic phase was separated. The aqueous phase was back-extracted with another 100 ml of dichloromethane, and the combined extracts were washed with saturated brine, dried over sodium sulfate and evaporated on a rotary evaporator to afford the crude product as a white solid, which was triturated with petroleum ether to furnish the pure product as colourless crystals after drying *in vacuo* at room temperature. Crystals suitable for diffraction were obtained by slow evaporation of a solution of the title compound in petroleum ether/dichloromethane (1:5 *v*/*v*) at room temperature.

### Refinement

All H atoms were positioned geometrically and refined using a riding model, with C—H = 0.95–0.98 Å, O—H = 0.84 Å, and with *U*_iso_(H) = 1.2*U*_eq_(C) or 1.5*U*_eq_(C, O) for methyl and hydroxyl H atoms.

### Crystal data

**Table:**

C_10_H_13_BrO	*Z* = 8
*M**_r_* = 229.11	*F*(000) = 928
Triclinic, *P*1	*D*_x_ = 1.497 Mg m^−^^3^
Hall symbol: -P 1	Mo *K*α radiation, λ = 0.71073 Å
*a* = 12.074 (2) Å	Cell parameters from 5648 reflections
*b* = 12.115 (2) Å	θ = 1.7–27.9°
*c* = 15.242 (3) Å	µ = 4.00 mm^−^^1^
α = 109.51 (3)°	*T* = 113 K
β = 103.52 (3)°	Block, colourless
γ = 90.70 (3)°	0.26 × 0.20 × 0.18 mm
*V* = 2033.2 (7) Å^3^	

### Data collection

**Table:**

Rigaku Saturn CCD area-detector diffractometer	7162 independent reflections
Radiation source: rotating anode	5048 reflections with *I* > 2σ(*I*)
confocal	*R*_int_ = 0.077
Detector resolution: 7.31 pixels mm^-1^	θ_max_ = 25.0°, θ_min_ = 1.5°
ω and φ scans	*h* = −14→14
Absorption correction: multi-scan (*CrystalClear*; Rigaku, 2007)	*k* = −14→14
*T*_min_ = 0.423, *T*_max_ = 0.533	*l* = −18→18
20930 measured reflections	

### Refinement

**Table:**

Refinement on *F*^2^	Secondary atom site location: difference Fourier map
Least-squares matrix: full	Hydrogen site location: inferred from neighbouring sites
*R*[*F*^2^ > 2σ(*F*^2^)] = 0.049	H-atom parameters constrained
*wR*(*F*^2^) = 0.102	*w* = 1/[σ^2^(*F*_o_^2^) + (0.0407*P*)^2^] where *P* = (*F*_o_^2^ + 2*F*_c_^2^)/3
*S* = 0.94	(Δ/σ)_max_ = 0.050
7162 reflections	Δρ_max_ = 1.36 e Å^−^^3^
450 parameters	Δρ_min_ = −1.05 e Å^−^^3^
0 restraints	Extinction correction: *SHELXL97* (Sheldrick, 2008), Fc^*^=kFc[1+0.001xFc^2^λ^3^/sin(2θ)]^-1/4^
Primary atom site location: structure-invariant direct methods	Extinction coefficient: 0.0140 (5)

### Special details

**Table:**

Geometry. All e.s.d.'s (except the e.s.d. in the dihedral angle between two l.s. planes) are estimated using the full covariance matrix. The cell e.s.d.'s are taken into account individually in the estimation of e.s.d.'s in distances, angles and torsion angles; correlations between e.s.d.'s in cell parameters are only used when they are defined by crystal symmetry. An approximate (isotropic) treatment of cell e.s.d.'s is used for estimating e.s.d.'s involving l.s. planes.
Refinement. Refinement of *F*^2^ against ALL reflections. The weighted *R*-factor *wR* and goodness of fit *S* are based on *F*^2^, conventional *R*-factors *R* are based on *F*, with *F* set to zero for negative *F*^2^. The threshold expression of *F*^2^ > σ(*F*^2^) is used only for calculating *R*-factors(gt) *etc*. and is not relevant to the choice of reflections for refinement. *R*-factors based on *F*^2^ are statistically about twice as large as those based on *F*, and *R*- factors based on ALL data will be even larger.

### Fractional atomic coordinates and isotropic or equivalent isotropic displacement parameters (Å^2^)

**Table:**

	*x*	*y*	*z*	*U*_iso_*/*U*_eq_	
Br1	1.20641 (4)	0.55935 (4)	0.04617 (3)	0.04051 (17)	
Br2	0.34327 (4)	0.53660 (4)	0.47320 (4)	0.04298 (17)	
Br3	0.48759 (4)	0.22317 (4)	0.56556 (4)	0.04416 (17)	
Br4	0.46999 (4)	0.38992 (5)	0.06139 (4)	0.05014 (19)	
O1	0.8786 (2)	0.9073 (2)	0.2449 (2)	0.0251 (7)	
H1	0.9348	0.9484	0.2444	0.038*	
O2	0.0633 (2)	0.0422 (2)	0.2459 (2)	0.0276 (7)	
H2	0.0242	0.0981	0.2422	0.041*	
O3	0.2597 (2)	−0.0834 (2)	0.7560 (2)	0.0264 (7)	
H3	0.2269	−0.0218	0.7606	0.040*	
O4	0.9259 (2)	0.2213 (2)	0.2509 (2)	0.0300 (7)	
H4	0.8695	0.1787	0.2492	0.045*	
C1	1.1312 (3)	0.6251 (3)	0.1462 (3)	0.0216 (10)	
C2	1.1757 (3)	0.6195 (3)	0.2360 (3)	0.0269 (10)	
H2A	1.2430	0.5819	0.2493	0.032*	
C3	1.1191 (3)	0.6705 (3)	0.3065 (3)	0.0282 (11)	
H3A	1.1490	0.6673	0.3689	0.034*	
C4	1.0203 (3)	0.7263 (3)	0.2901 (3)	0.0214 (10)	
C5	0.9755 (3)	0.7312 (3)	0.1972 (3)	0.0173 (9)	
C6	1.0333 (3)	0.6798 (3)	0.1268 (3)	0.0202 (9)	
H6	1.0047	0.6823	0.0640	0.024*	
C7	0.8679 (3)	0.7921 (3)	0.1722 (3)	0.0203 (9)	
C8	0.7608 (3)	0.7233 (3)	0.1740 (3)	0.0279 (10)	
H8A	0.7714	0.7149	0.2371	0.042*	
H8B	0.7490	0.6452	0.1240	0.042*	
H8C	0.6938	0.7661	0.1619	0.042*	
C9	0.8479 (3)	0.8080 (4)	0.0753 (3)	0.0284 (11)	
H9A	0.7817	0.8527	0.0663	0.043*	
H9B	0.8333	0.7307	0.0241	0.043*	
H9C	0.9159	0.8508	0.0729	0.043*	
C10	0.9676 (4)	0.7776 (4)	0.3746 (3)	0.0337 (11)	
H10A	0.8965	0.7297	0.3640	0.051*	
H10B	0.9510	0.8584	0.3806	0.051*	
H10C	1.0214	0.7778	0.4338	0.051*	
C11	0.3139 (3)	0.3892 (3)	0.3709 (3)	0.0257 (10)	
C12	0.3457 (3)	0.3765 (4)	0.2870 (3)	0.0324 (12)	
H12	0.3853	0.4397	0.2796	0.039*	
C13	0.3173 (3)	0.2680 (4)	0.2141 (3)	0.0293 (11)	
H13	0.3386	0.2580	0.1559	0.035*	
C14	0.2595 (3)	0.1730 (4)	0.2214 (3)	0.0235 (10)	
C15	0.2295 (3)	0.1867 (3)	0.3085 (3)	0.0183 (9)	
C16	0.2580 (3)	0.2966 (3)	0.3821 (3)	0.0207 (9)	
H16	0.2384	0.3076	0.4411	0.025*	
C17	0.1661 (3)	0.0865 (3)	0.3240 (3)	0.0220 (10)	
C18	0.2385 (3)	−0.0160 (4)	0.3225 (3)	0.0309 (11)	
H18A	0.1957	−0.0776	0.3331	0.046*	
H18B	0.3096	0.0115	0.3735	0.046*	
H18C	0.2567	−0.0479	0.2600	0.046*	
C19	0.1280 (4)	0.1253 (4)	0.4174 (3)	0.0323 (11)	
H19A	0.0810	0.1911	0.4196	0.049*	
H19B	0.1955	0.1504	0.4722	0.049*	
H19C	0.0831	0.0594	0.4205	0.049*	
C20	0.2293 (4)	0.0606 (4)	0.1338 (3)	0.0371 (12)	
H20A	0.2312	0.0788	0.0761	0.056*	
H20B	0.1524	0.0263	0.1267	0.056*	
H20C	0.2849	0.0043	0.1421	0.056*	
C21	0.4838 (3)	0.1617 (4)	0.6641 (3)	0.0239 (10)	
C22	0.5489 (3)	0.2199 (4)	0.7562 (3)	0.0259 (10)	
H22	0.5965	0.2900	0.7710	0.031*	
C23	0.5432 (3)	0.1731 (4)	0.8269 (3)	0.0253 (10)	
H23	0.5887	0.2124	0.8904	0.030*	
C24	0.4740 (3)	0.0717 (3)	0.8092 (3)	0.0211 (9)	
C25	0.4076 (3)	0.0124 (3)	0.7140 (3)	0.0185 (9)	
C26	0.4141 (3)	0.0585 (3)	0.6426 (3)	0.0219 (10)	
H26	0.3705	0.0191	0.5785	0.026*	
C27	0.3283 (3)	−0.1005 (3)	0.6868 (3)	0.0215 (10)	
C28	0.2452 (3)	−0.1334 (4)	0.5874 (3)	0.0329 (11)	
H28A	0.1945	−0.2035	0.5756	0.049*	
H28B	0.2885	−0.1500	0.5383	0.049*	
H28C	0.1993	−0.0679	0.5847	0.049*	
C29	0.3978 (4)	−0.2032 (3)	0.6918 (3)	0.0315 (11)	
H29A	0.4465	−0.1846	0.7571	0.047*	
H29B	0.4458	−0.2172	0.6462	0.047*	
H29C	0.3458	−0.2739	0.6753	0.047*	
C30	0.4746 (4)	0.0326 (4)	0.8945 (3)	0.0331 (11)	
H30A	0.4922	−0.0494	0.8791	0.050*	
H30B	0.3992	0.0394	0.9084	0.050*	
H30C	0.5329	0.0827	0.9509	0.050*	
C31	0.6097 (3)	0.4163 (4)	0.1563 (3)	0.0269 (10)	
C32	0.6225 (4)	0.5019 (4)	0.2444 (3)	0.0348 (12)	
H32	0.5627	0.5500	0.2583	0.042*	
C33	0.7253 (4)	0.5166 (4)	0.3131 (3)	0.0356 (12)	
H33	0.7355	0.5774	0.3737	0.043*	
C34	0.8145 (4)	0.4461 (4)	0.2972 (3)	0.0268 (10)	
C35	0.8002 (3)	0.3588 (3)	0.2055 (3)	0.0201 (9)	
C36	0.6960 (3)	0.3454 (4)	0.1365 (3)	0.0225 (10)	
H36	0.6846	0.2863	0.0748	0.027*	
C37	0.8929 (3)	0.2772 (4)	0.1789 (3)	0.0257 (10)	
C38	1.0005 (3)	0.3467 (4)	0.1813 (3)	0.0326 (11)	
H38A	1.0330	0.4019	0.2467	0.049*	
H38B	0.9808	0.3906	0.1371	0.049*	
H38C	1.0568	0.2924	0.1615	0.049*	
C39	0.8532 (4)	0.1783 (4)	0.0817 (3)	0.0359 (12)	
H39A	0.9139	0.1259	0.0720	0.054*	
H39B	0.8359	0.2120	0.0304	0.054*	
H39C	0.7843	0.1334	0.0802	0.054*	
C40	0.9194 (4)	0.4674 (4)	0.3803 (3)	0.0411 (13)	
H40A	0.9771	0.5215	0.3757	0.062*	
H40B	0.9510	0.3925	0.3775	0.062*	
H40C	0.8980	0.5020	0.4412	0.062*	

### Atomic displacement parameters (Å^2^)

**Table:**

	*U*^11^	*U*^22^	*U*^33^	*U*^12^	*U*^13^	*U*^23^
Br1	0.0292 (3)	0.0346 (3)	0.0496 (3)	0.0067 (2)	0.0175 (2)	−0.0012 (3)
Br2	0.0500 (3)	0.0189 (3)	0.0533 (4)	−0.0039 (2)	0.0047 (3)	0.0098 (2)
Br3	0.0583 (4)	0.0459 (3)	0.0442 (3)	0.0046 (3)	0.0202 (3)	0.0312 (3)
Br4	0.0220 (3)	0.0828 (4)	0.0657 (4)	0.0112 (3)	0.0060 (3)	0.0548 (3)
O1	0.0231 (16)	0.0154 (16)	0.0339 (18)	0.0017 (12)	0.0101 (14)	0.0029 (14)
O2	0.0225 (16)	0.0203 (16)	0.0364 (18)	0.0026 (12)	−0.0010 (14)	0.0108 (15)
O3	0.0236 (17)	0.0216 (17)	0.0432 (19)	0.0088 (13)	0.0158 (15)	0.0178 (15)
O4	0.0218 (16)	0.0284 (18)	0.045 (2)	0.0021 (13)	0.0024 (15)	0.0235 (15)
C1	0.018 (2)	0.017 (2)	0.028 (3)	−0.0004 (17)	0.0066 (19)	0.005 (2)
C2	0.017 (2)	0.019 (2)	0.045 (3)	0.0010 (18)	0.002 (2)	0.015 (2)
C3	0.027 (3)	0.029 (3)	0.031 (3)	−0.003 (2)	−0.003 (2)	0.020 (2)
C4	0.021 (2)	0.021 (2)	0.021 (2)	−0.0043 (18)	0.0016 (19)	0.0086 (19)
C5	0.016 (2)	0.013 (2)	0.023 (2)	−0.0017 (16)	0.0028 (18)	0.0085 (18)
C6	0.021 (2)	0.015 (2)	0.020 (2)	−0.0013 (17)	0.0021 (19)	0.0044 (19)
C7	0.018 (2)	0.015 (2)	0.027 (3)	0.0045 (17)	0.0034 (19)	0.006 (2)
C8	0.022 (2)	0.024 (2)	0.036 (3)	0.0010 (18)	0.003 (2)	0.011 (2)
C9	0.030 (3)	0.037 (3)	0.024 (3)	0.010 (2)	0.006 (2)	0.017 (2)
C10	0.040 (3)	0.035 (3)	0.026 (3)	0.003 (2)	0.008 (2)	0.011 (2)
C11	0.024 (2)	0.017 (2)	0.035 (3)	0.0005 (18)	0.000 (2)	0.013 (2)
C12	0.019 (2)	0.035 (3)	0.060 (3)	0.009 (2)	0.014 (2)	0.034 (3)
C13	0.025 (2)	0.045 (3)	0.034 (3)	0.017 (2)	0.014 (2)	0.030 (3)
C14	0.018 (2)	0.028 (3)	0.027 (3)	0.0094 (18)	0.0054 (19)	0.013 (2)
C15	0.012 (2)	0.022 (2)	0.025 (2)	0.0068 (17)	0.0049 (18)	0.012 (2)
C16	0.021 (2)	0.021 (2)	0.025 (2)	0.0061 (18)	0.0068 (19)	0.013 (2)
C17	0.022 (2)	0.017 (2)	0.028 (3)	0.0024 (18)	0.008 (2)	0.009 (2)
C18	0.030 (3)	0.024 (3)	0.042 (3)	0.0051 (19)	0.005 (2)	0.018 (2)
C19	0.034 (3)	0.032 (3)	0.038 (3)	−0.002 (2)	0.016 (2)	0.017 (2)
C20	0.043 (3)	0.047 (3)	0.023 (3)	0.015 (2)	0.013 (2)	0.010 (2)
C21	0.027 (2)	0.030 (3)	0.025 (3)	0.008 (2)	0.014 (2)	0.017 (2)
C22	0.019 (2)	0.024 (2)	0.038 (3)	0.0034 (18)	0.008 (2)	0.013 (2)
C23	0.022 (2)	0.028 (3)	0.022 (2)	0.0008 (19)	0.0010 (19)	0.006 (2)
C24	0.021 (2)	0.027 (2)	0.020 (2)	0.0048 (18)	0.0071 (19)	0.012 (2)
C25	0.018 (2)	0.019 (2)	0.021 (2)	0.0081 (17)	0.0044 (18)	0.0093 (19)
C26	0.023 (2)	0.022 (2)	0.022 (2)	0.0028 (18)	0.0067 (19)	0.008 (2)
C27	0.020 (2)	0.020 (2)	0.024 (2)	0.0034 (18)	0.0054 (19)	0.007 (2)
C28	0.031 (3)	0.029 (3)	0.032 (3)	−0.004 (2)	0.000 (2)	0.009 (2)
C29	0.033 (3)	0.022 (2)	0.041 (3)	0.007 (2)	0.013 (2)	0.010 (2)
C30	0.036 (3)	0.046 (3)	0.021 (3)	0.001 (2)	0.002 (2)	0.019 (2)
C31	0.023 (2)	0.031 (3)	0.032 (3)	0.001 (2)	0.008 (2)	0.018 (2)
C32	0.036 (3)	0.029 (3)	0.056 (3)	0.012 (2)	0.023 (3)	0.028 (3)
C33	0.055 (3)	0.018 (3)	0.038 (3)	0.004 (2)	0.024 (3)	0.007 (2)
C34	0.034 (3)	0.019 (2)	0.025 (3)	−0.0076 (19)	0.004 (2)	0.007 (2)
C35	0.023 (2)	0.015 (2)	0.023 (2)	0.0002 (17)	0.0035 (19)	0.0101 (19)
C36	0.023 (2)	0.028 (3)	0.024 (2)	0.0050 (19)	0.010 (2)	0.015 (2)
C37	0.020 (2)	0.028 (3)	0.033 (3)	0.0036 (19)	0.004 (2)	0.016 (2)
C38	0.027 (3)	0.032 (3)	0.045 (3)	0.004 (2)	0.013 (2)	0.019 (2)
C39	0.035 (3)	0.028 (3)	0.043 (3)	0.008 (2)	0.015 (2)	0.006 (2)
C40	0.051 (3)	0.038 (3)	0.024 (3)	−0.011 (2)	−0.005 (2)	0.007 (2)

### Geometric parameters (Å, °)

**Table:**

Br1—C1	1.907 (4)	C19—H19A	0.9800
Br2—C11	1.901 (4)	C19—H19B	0.9800
Br3—C21	1.896 (4)	C19—H19C	0.9800
Br4—C31	1.891 (4)	C20—H20A	0.9800
O1—C7	1.445 (4)	C20—H20B	0.9800
O1—H1	0.8400	C20—H20C	0.9800
O2—C17	1.453 (4)	C21—C22	1.376 (5)
O2—H2	0.8400	C21—C26	1.396 (5)
O3—C27	1.451 (4)	C22—C23	1.388 (5)
O3—H3	0.8400	C22—H22	0.9500
O4—C37	1.456 (5)	C23—C24	1.389 (5)
O4—H4	0.8400	C23—H23	0.9500
C1—C2	1.374 (6)	C24—C25	1.417 (5)
C1—C6	1.382 (5)	C24—C30	1.524 (5)
C2—C3	1.383 (5)	C25—C26	1.395 (5)
C2—H2A	0.9500	C25—C27	1.538 (5)
C3—C4	1.391 (5)	C26—H26	0.9500
C3—H3A	0.9500	C27—C29	1.522 (5)
C4—C5	1.414 (5)	C27—C28	1.529 (5)
C4—C10	1.520 (5)	C28—H28A	0.9800
C5—C6	1.393 (5)	C28—H28B	0.9800
C5—C7	1.535 (5)	C28—H28C	0.9800
C6—H6	0.9500	C29—H29A	0.9800
C7—C9	1.517 (5)	C29—H29B	0.9800
C7—C8	1.541 (5)	C29—H29C	0.9800
C8—H8A	0.9800	C30—H30A	0.9800
C8—H8B	0.9800	C30—H30B	0.9800
C8—H8C	0.9800	C30—H30C	0.9800
C9—H9A	0.9800	C31—C32	1.369 (6)
C9—H9B	0.9800	C31—C36	1.382 (5)
C9—H9C	0.9800	C32—C33	1.390 (6)
C10—H10A	0.9800	C32—H32	0.9500
C10—H10B	0.9800	C33—C34	1.397 (6)
C10—H10C	0.9800	C33—H33	0.9500
C11—C16	1.381 (5)	C34—C35	1.413 (5)
C11—C12	1.381 (5)	C34—C40	1.516 (6)
C12—C13	1.383 (6)	C35—C36	1.407 (5)
C12—H12	0.9500	C35—C37	1.538 (5)
C13—C14	1.389 (5)	C36—H36	0.9500
C13—H13	0.9500	C37—C38	1.527 (5)
C14—C15	1.413 (5)	C37—C39	1.526 (5)
C14—C20	1.522 (5)	C38—H38A	0.9800
C15—C16	1.400 (5)	C38—H38B	0.9800
C15—C17	1.539 (5)	C38—H38C	0.9800
C16—H16	0.9500	C39—H39A	0.9800
C17—C19	1.525 (5)	C39—H39B	0.9800
C17—C18	1.525 (5)	C39—H39C	0.9800
C18—H18A	0.9800	C40—H40A	0.9800
C18—H18B	0.9800	C40—H40B	0.9800
C18—H18C	0.9800	C40—H40C	0.9800
			
C7—O1—H1	109.5	H20A—C20—H20C	109.5
C17—O2—H2	109.5	H20B—C20—H20C	109.5
C27—O3—H3	109.5	C22—C21—C26	120.8 (4)
C37—O4—H4	109.5	C22—C21—Br3	119.6 (3)
C2—C1—C6	121.2 (4)	C26—C21—Br3	119.6 (3)
C2—C1—Br1	120.0 (3)	C21—C22—C23	118.1 (4)
C6—C1—Br1	118.8 (3)	C21—C22—H22	120.9
C1—C2—C3	117.7 (4)	C23—C22—H22	120.9
C1—C2—H2A	121.1	C24—C23—C22	123.1 (4)
C3—C2—H2A	121.1	C24—C23—H23	118.5
C2—C3—C4	123.0 (4)	C22—C23—H23	118.5
C2—C3—H3A	118.5	C23—C24—C25	118.2 (4)
C4—C3—H3A	118.5	C23—C24—C30	117.1 (4)
C3—C4—C5	118.7 (4)	C25—C24—C30	124.6 (4)
C3—C4—C10	116.8 (4)	C26—C25—C24	118.8 (4)
C5—C4—C10	124.5 (4)	C26—C25—C27	118.9 (3)
C6—C5—C4	117.9 (3)	C24—C25—C27	122.3 (3)
C6—C5—C7	119.5 (3)	C25—C26—C21	121.0 (4)
C4—C5—C7	122.7 (3)	C25—C26—H26	119.5
C1—C6—C5	121.6 (4)	C21—C26—H26	119.5
C1—C6—H6	119.2	O3—C27—C29	107.0 (3)
C5—C6—H6	119.2	O3—C27—C28	107.1 (3)
O1—C7—C9	107.8 (3)	C29—C27—C28	109.5 (3)
O1—C7—C5	108.8 (3)	O3—C27—C25	109.1 (3)
C9—C7—C5	113.9 (3)	C29—C27—C25	110.7 (3)
O1—C7—C8	106.9 (3)	C28—C27—C25	113.3 (3)
C9—C7—C8	108.6 (3)	C27—C28—H28A	109.5
C5—C7—C8	110.6 (3)	C27—C28—H28B	109.5
C7—C8—H8A	109.5	H28A—C28—H28B	109.5
C7—C8—H8B	109.5	C27—C28—H28C	109.5
H8A—C8—H8B	109.5	H28A—C28—H28C	109.5
C7—C8—H8C	109.5	H28B—C28—H28C	109.5
H8A—C8—H8C	109.5	C27—C29—H29A	109.5
H8B—C8—H8C	109.5	C27—C29—H29B	109.5
C7—C9—H9A	109.5	H29A—C29—H29B	109.5
C7—C9—H9B	109.5	C27—C29—H29C	109.5
H9A—C9—H9B	109.5	H29A—C29—H29C	109.5
C7—C9—H9C	109.5	H29B—C29—H29C	109.5
H9A—C9—H9C	109.5	C24—C30—H30A	109.5
H9B—C9—H9C	109.5	C24—C30—H30B	109.5
C4—C10—H10A	109.5	H30A—C30—H30B	109.5
C4—C10—H10B	109.5	C24—C30—H30C	109.5
H10A—C10—H10B	109.5	H30A—C30—H30C	109.5
C4—C10—H10C	109.5	H30B—C30—H30C	109.5
H10A—C10—H10C	109.5	C32—C31—C36	121.0 (4)
H10B—C10—H10C	109.5	C32—C31—Br4	119.8 (3)
C16—C11—C12	121.2 (4)	C36—C31—Br4	119.2 (3)
C16—C11—Br2	118.3 (3)	C31—C32—C33	118.1 (4)
C12—C11—Br2	120.5 (3)	C31—C32—H32	121.0
C11—C12—C13	117.3 (4)	C33—C32—H32	121.0
C11—C12—H12	121.3	C32—C33—C34	123.2 (4)
C13—C12—H12	121.3	C32—C33—H33	118.4
C12—C13—C14	123.5 (4)	C34—C33—H33	118.4
C12—C13—H13	118.3	C33—C34—C35	117.9 (4)
C14—C13—H13	118.3	C33—C34—C40	117.6 (4)
C13—C14—C15	118.5 (4)	C35—C34—C40	124.4 (4)
C13—C14—C20	117.8 (4)	C36—C35—C34	118.3 (4)
C15—C14—C20	123.7 (4)	C36—C35—C37	119.0 (3)
C16—C15—C14	117.9 (4)	C34—C35—C37	122.7 (4)
C16—C15—C17	119.6 (3)	C31—C36—C35	121.5 (4)
C14—C15—C17	122.5 (4)	C31—C36—H36	119.2
C11—C16—C15	121.5 (4)	C35—C36—H36	119.2
C11—C16—H16	119.2	O4—C37—C38	106.8 (3)
C15—C16—H16	119.2	O4—C37—C39	106.7 (3)
O2—C17—C19	106.9 (3)	C38—C37—C39	109.5 (3)
O2—C17—C18	107.5 (3)	O4—C37—C35	108.4 (3)
C19—C17—C18	108.2 (3)	C38—C37—C35	111.2 (3)
O2—C17—C15	108.6 (3)	C39—C37—C35	113.9 (3)
C19—C17—C15	113.5 (3)	C37—C38—H38A	109.5
C18—C17—C15	111.8 (3)	C37—C38—H38B	109.5
C17—C18—H18A	109.5	H38A—C38—H38B	109.5
C17—C18—H18B	109.5	C37—C38—H38C	109.5
H18A—C18—H18B	109.5	H38A—C38—H38C	109.5
C17—C18—H18C	109.5	H38B—C38—H38C	109.5
H18A—C18—H18C	109.5	C37—C39—H39A	109.5
H18B—C18—H18C	109.5	C37—C39—H39B	109.5
C17—C19—H19A	109.5	H39A—C39—H39B	109.5
C17—C19—H19B	109.5	C37—C39—H39C	109.5
H19A—C19—H19B	109.5	H39A—C39—H39C	109.5
C17—C19—H19C	109.5	H39B—C39—H39C	109.5
H19A—C19—H19C	109.5	C34—C40—H40A	109.5
H19B—C19—H19C	109.5	C34—C40—H40B	109.5
C14—C20—H20A	109.5	H40A—C40—H40B	109.5
C14—C20—H20B	109.5	C34—C40—H40C	109.5
H20A—C20—H20B	109.5	H40A—C40—H40C	109.5
C14—C20—H20C	109.5	H40B—C40—H40C	109.5
			
C6—C1—C2—C3	0.0 (6)	C26—C21—C22—C23	−0.1 (6)
Br1—C1—C2—C3	178.8 (3)	Br3—C21—C22—C23	179.3 (3)
C1—C2—C3—C4	0.0 (6)	C21—C22—C23—C24	−0.8 (6)
C2—C3—C4—C5	0.0 (6)	C22—C23—C24—C25	1.0 (6)
C2—C3—C4—C10	179.7 (4)	C22—C23—C24—C30	−178.6 (4)
C3—C4—C5—C6	−0.2 (5)	C23—C24—C25—C26	−0.3 (5)
C10—C4—C5—C6	−179.9 (4)	C30—C24—C25—C26	179.3 (4)
C3—C4—C5—C7	−179.2 (3)	C23—C24—C25—C27	−179.9 (3)
C10—C4—C5—C7	1.1 (6)	C30—C24—C25—C27	−0.4 (6)
C2—C1—C6—C5	−0.2 (6)	C24—C25—C26—C21	−0.6 (6)
Br1—C1—C6—C5	−178.9 (3)	C27—C25—C26—C21	179.1 (3)
C4—C5—C6—C1	0.2 (5)	C22—C21—C26—C25	0.8 (6)
C7—C5—C6—C1	179.3 (3)	Br3—C21—C26—C25	−178.7 (3)
C6—C5—C7—O1	−130.5 (4)	C26—C25—C27—O3	−132.1 (3)
C4—C5—C7—O1	48.5 (5)	C24—C25—C27—O3	47.6 (5)
C6—C5—C7—C9	−10.3 (5)	C26—C25—C27—C29	110.5 (4)
C4—C5—C7—C9	168.7 (4)	C24—C25—C27—C29	−69.9 (5)
C6—C5—C7—C8	112.4 (4)	C26—C25—C27—C28	−12.9 (5)
C4—C5—C7—C8	−68.6 (5)	C24—C25—C27—C28	166.7 (4)
C16—C11—C12—C13	1.1 (6)	C36—C31—C32—C33	0.8 (6)
Br2—C11—C12—C13	−177.4 (3)	Br4—C31—C32—C33	178.4 (3)
C11—C12—C13—C14	0.0 (6)	C31—C32—C33—C34	−1.8 (6)
C12—C13—C14—C15	−1.2 (6)	C32—C33—C34—C35	2.4 (6)
C12—C13—C14—C20	177.3 (4)	C32—C33—C34—C40	−176.7 (4)
C13—C14—C15—C16	1.2 (5)	C33—C34—C35—C36	−1.9 (6)
C20—C14—C15—C16	−177.2 (4)	C40—C34—C35—C36	177.1 (4)
C13—C14—C15—C17	−179.6 (3)	C33—C34—C35—C37	179.1 (4)
C20—C14—C15—C17	2.0 (6)	C40—C34—C35—C37	−1.9 (6)
C12—C11—C16—C15	−1.0 (6)	C32—C31—C36—C35	−0.4 (6)
Br2—C11—C16—C15	177.5 (3)	Br4—C31—C36—C35	−178.0 (3)
C14—C15—C16—C11	−0.2 (6)	C34—C35—C36—C31	1.0 (6)
C17—C15—C16—C11	−179.4 (4)	C37—C35—C36—C31	−180.0 (4)
C16—C15—C17—O2	125.9 (4)	C36—C35—C37—O4	−126.0 (4)
C14—C15—C17—O2	−53.3 (5)	C34—C35—C37—O4	53.0 (5)
C16—C15—C17—C19	7.1 (5)	C36—C35—C37—C38	116.8 (4)
C14—C15—C17—C19	−172.1 (4)	C34—C35—C37—C38	−64.2 (5)
C16—C15—C17—C18	−115.7 (4)	C36—C35—C37—C39	−7.4 (5)
C14—C15—C17—C18	65.1 (5)	C34—C35—C37—C39	171.6 (4)

### Hydrogen-bond geometry (Å, °)

**Table:**

*D*—H···*A*	*D*—H	H···*A*	*D*···*A*	*D*—H···*A*
O1—H1···O2^i^	0.84	1.90	2.742 (4)	179
O2—H2···O4^ii^	0.84	1.91	2.739 (4)	170
O3—H3···O1^iii^	0.84	1.90	2.727 (4)	167
O4—H4···O3^iv^	0.84	1.90	2.740 (4)	179

Symmetry codes: (i) *x*+1, *y*+1, *z*; (ii) *x*−1, *y*, *z*; (iii) −*x*+1, −*y*+1, −*z*+1; (iv) −*x*+1, −*y*, −*z*+1.
